# p300-mediated acetylation of COMMD1 regulates its stability, and the ubiquitylation and nucleolar translocation of the RelA NF-κB subunit

**DOI:** 10.1242/jcs.149328

**Published:** 2014-09-01

**Authors:** Andrew O'Hara, James Simpson, Pierre Morin, Carolyn J. Loveridge, Ann C. Williams, Sonia M. Novo, Lesley A. Stark

**Affiliations:** 1 Edinburgh Cancer Research Centre, IGMM, University of Edinburgh, Western General Hospital, Crewe Road, Edinburgh EH4 2XU, UK; 2 Colorectal Tumour Biology Group, School of Cellular and Molecular Medicine, University of Bristol, Bristol BS8 1TH, UK

**Keywords:** Aspirin, Chemoprevention, NF-κB, Apoptosis, Acetylation, Nucleoli, Nucleolus, COMMD1, p300, XIAP

## Abstract

Nucleolar sequestration of the RelA subunit of nuclear factor (NF)-κB is an important mechanism for regulating NF-κB transcriptional activity. Ubiquitylation, facilitated by COMMD1 (also known as MURR1), acts as a crucial nucleolar-targeting signal for RelA, but how this ubiquitylation is regulated, and how it differs from cytokine-mediated ubiquitylation, which causes proteasomal degradation of RelA, is poorly understood. Here, we report a new role for p300 (also known as EP300) in controlling stimulus-specific ubiquitylation of RelA, through modulation of COMMD1. We show that p300 is required for stress-mediated ubiquitylation and nucleolar translocation of RelA, but that this effect is indirect. We also demonstrate that COMMD1 is acetylated by p300 and that acetylation protects COMMD1 from XIAP-mediated proteosomal degradation. Furthermore, we demonstrate that COMMD1 acetylation is enhanced by aspirin-mediated stress, and that this acetylation is absolutely required for the protein to bind RelA under these conditions. In contrast, tumour necrosis factor (TNF) has no effect on COMMD1 acetylation. Finally, we demonstrate these findings have relevance in a whole tissue setting. These data offer a new paradigm for the regulation of NF-κB transcriptional activity, and the multiple other pathways controlled by COMMD1.

## INTRODUCTION

The nuclear factor (NF)-κB transcription factor plays a central role in many processes, including inflammation and stress response (Perkins and Gilmore, 2006). The most recognised mechanism for regulating its activity is cytoplasmic to nuclear translocation (Karin, 1999). However, once in the nucleus, NF-κB is modulated by a plethora of co-activators, repressors and post-transcriptional modifications (Huang et al., 2010; Yang et al., 2010). These nuclear regulatory pathways are important because they determine the downstream consequences of stimulating NF-κB. However, they remain poorly understood.

One mechanism that regulates NF-κB activity in the nucleus is the distribution of the RelA (also known as p65) subunit (Loveridge et al., 2008; Stark and Dunlop, 2005). Inflammatory cytokines, such as tumour necrosis factor (TNF), cause nucleoplasmic accumulation of RelA, which activates NF-κB-driven transcription (Perkins and Gilmore, 2006), whereas specific stress stimuli (including UV-C radiation, serum deprivation and aspirin) cause nucleolar accumulation of RelA, which represses NF-κB-driven transcription (Khandelwal et al., 2011; Stark and Dunlop, 2005; Thoms et al., 2007).

Understanding of the pathways that regulate RelA nuclear distribution is limited, but work from our group has previously identified ubiquitylation as crucial (Thoms et al., 2010). We have demonstrated that stress stimuli cause polyubiquitylation of RelA and increased levels of the ubiquitin ligase co-factor COMMD1. Furthermore, we have demonstrated that COMMD1-facilitated ubiquitylation is essential for nucleoplasmic–nucleolar translocation of RelA. TNF also induces COMMD1-dependent RelA ubiquitylation, but this targets the protein for degradation rather than nucleolar translocation, suggesting that different ubiquitylation pathways and/or signatures might be involved (Colleran et al., 2013; Saccani et al., 2004).

Acetylation also plays an essential role in regulating nuclear RelA (Chen and Greene, 2004) and previous studies have suggested that this inhibits TNF-mediated ubiquitylation (Li et al., 2012). However, the role of acetylation and deacetylation in stress-induced ubiquitylation and nucleolar translocation of RelA is unknown.

Here, we report that an acetylation event does regulate the nucleolar translocation of RelA. We report that p300 (also known as EP300) acetylates COMMD1 and that this acetylation protects it from degradation. We also demonstrate that COMMD1 acetylation is enhanced in response to aspirin-mediated stress, and that this acetylation is required for the subsequent interaction with RelA. In contrast, TNF has no effect on COMMD1 acetylation. These data not only have relevance to the regulation of nuclear NF-κB activity, but also to the multiple other pathways modulated by the pleotropic protein COMMD1.

## RESULTS AND DISCUSSION

### p300 is required for the ubiquitylation and nucleolar translocation of RelA

**Fig. 1.**
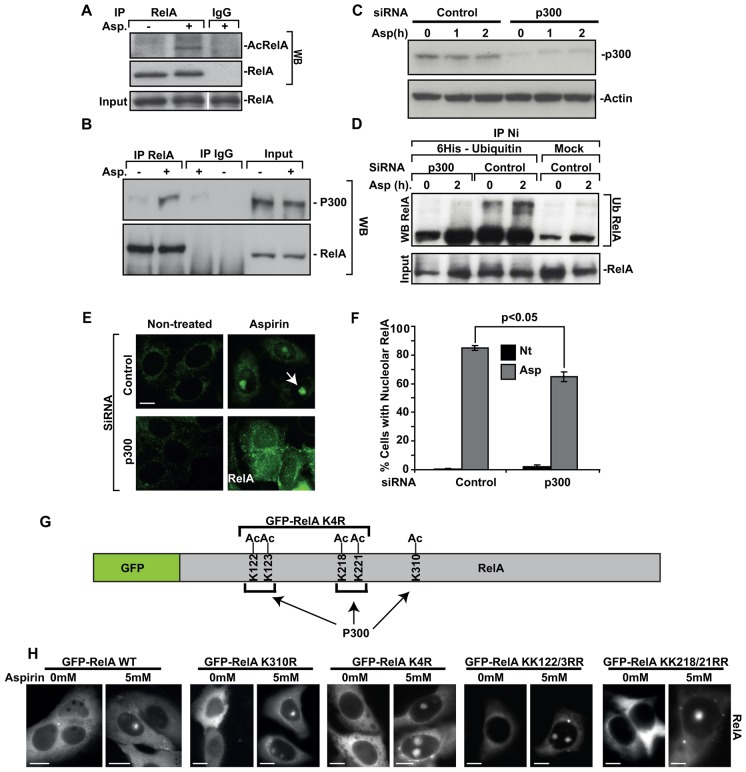
**p300 is required for the ubiquitylation and nucleolar translocation of RelA.** (A,B) SW480-GFP-RelA cells were exposed to 0 (−) or 5 mM (+) aspirin (Asp, 16 h), RelA was immunoprecipitated (IP), then recovered proteins were analysed by immunoblot (WB) for (A) acetylated RelA (AcRelA; anti-AcRelAK310 antibody) or (B) p300. Stripped gels were re-probed for RelA. Input levels of protein are shown. Rabbit IgG acts as a control. (C–F) SW480 cells were transfected with control (scrambled) or p300 siRNA, then treated with aspirin (C,D, 10 mM, times indicated; E,F, 5 mM, 16 h). (C) Immunoblot for p300. (D) Cells were co-transfected with His_6_–ubiquitin. Ubiquitylated RelA was analysed in lysates using nickel (Ni) agarose bead precipitation and immunoblotting for RelA. (E,F) Immunomicrographs demonstrating the localisation of RelA. The arrow indicates a nucleolus. The number of cells showing nucleolar RelA was determined in >200 cells from more than five fields. Data shown are the mean±s.e.m. *n* = 3. *P<0.05 (Student's *t*-test). (G,H) Diagram showing p300 acetylated lysine residues that were mutated to arginine residues. Live-cell imaging demonstrates nucleolar localisation of mutants. Scale bar: 10 µm.

Previous reports have suggested that RelA deacetylation is a pre-requisite for TNF-mediated ubiquitylation (Li et al., 2012). However, here, we found that aspirin-mediated ubiquitylation of RelA (aspirin is used as a model stress stimuli) is paralleled by increased acetylation of RelA and increased binding to the acetyltransferase p300 (Fig. 1A,B). We also found that p300 depletion abrogated aspirin-mediated ubiquitylation and nucleoplasmic to nucleolar translocation of RelA (Fig. 1C–F). However, site-directed mutagenesis revealed that all the identified p300 acetylation sites (K122, K123, K218 K221 and K310) within the first 311 amino acids of RelA [the minimal required for nucleolar localisation (Stark and Dunlop, 2005)] are dispensable for nucleolar transport of the protein (Fig. 1G–H).

These data would suggest that p300 is essential for the ubiquitylation of RelA associated with nucleolar translocation, but that this effect is indirect. As K310 and K123 are required for TNF-mediated ubiquitylation, the data would also suggest that the lysine residues important for stress and cytokine response differ (Li et al., 2012). Work is currently underway to define RelA residues required for stress-mediated ubiquitylation and nucleolar translocation of RelA. Here, we focus on essential acetylation events.

### p300 acetylates COMMD1 and regulates the stability of the protein

**Fig. 2.**
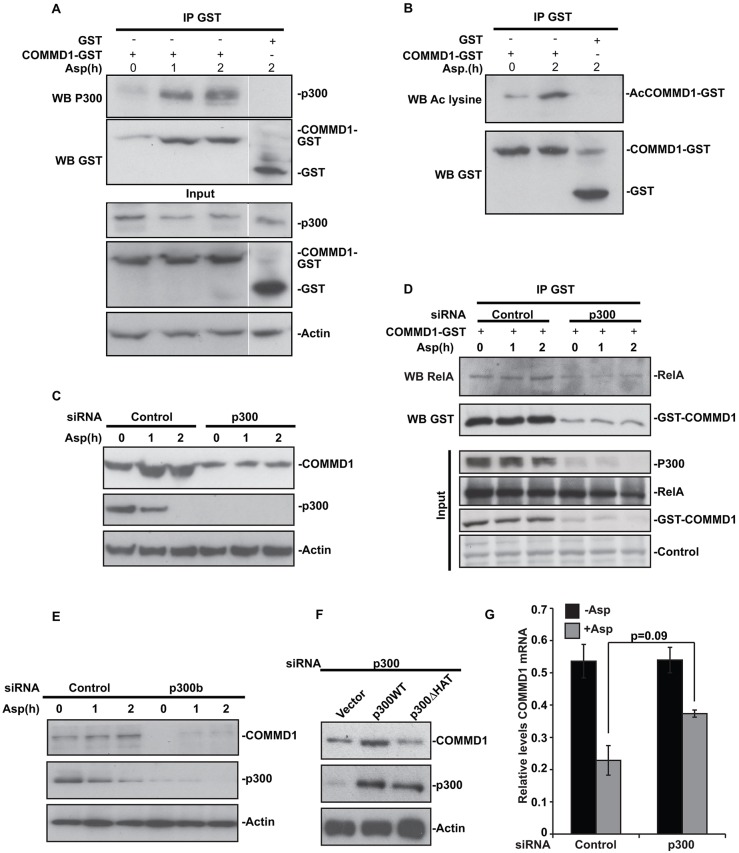
**p300 acetylates and regulates the stability of COMMD1.** (A,B,D) SW480 cells were transfected with COMMD–GST or GST alone (A,B), or together with control or p300 siRNA (D). Following aspirin (Asp) (10 mM, indicated times) treatment, GST-tagged proteins were isolated from whole-cell lysates using glutathione–agarose. Precipitated proteins were subjected to western blotting (WB) for (A) p300, (B) acetylated (Ac) lysine or (D) RelA. Gels were re-probed with GST to confirm equal GST–COMMD1 pulldown. (A,D) Input levels of indicated proteins are shown. Control, non-specific band. (C,E,F) Cells were transfected with control, p300 or p300b siRNA then (C,E) treated with aspirin (10 mM) for indicated times or (F) co-transfected with the plasmids indicated. Immunoblots show COMMD1 and p300 levels. Actin controls for loading. (G) Cells were transfected with control or p300 siRNA, treated with aspirin (5 mM, 16 h) then RNA was isolated. Real-time PCR was used to quantify COMMD1 transcripts relative to GAPDH control. The mean±s.e.m of three independent experiments is shown.

Instead of RelA, we considered the possibility that p300 modulates COMMD1. Indeed, immunoprecipitation indicated that COMMD1 interacts with p300, that this interaction is greatly enhanced upon aspirin exposure (despite a decrease in total cellular levels of p300) and that this is associated with COMMD1 acetylation (Fig. 2A,B). To determine the role of this acetylation in nucleolar transport of RelA we firstly utilised a small interfering RNA (siRNA) approach. Surprisingly, we found that depletion of p300 alone caused a substantial reduction in basal and induced levels of COMMD1 (Fig. 2C) and thus, abrogated the COMMD1–RelA interaction (Fig. 2D). Use of an independent siRNA confirmed the substantial depletion of COMMD1 upon p300 knockdown (Fig. 2E). Furthermore, overexpression studies indicated this could be rescued by expression of wild-type p300, but not an acetylation defective mutant [deleted for the histone acetyltransferase domain (ΔHAT)] (Dornan et al., 2004) (Fig. 2F).

Given that p300 acts as a transcriptional co-activator, we considered it might be required for COMMD1 transcription. However, qRT-PCR indicated p300 depletion had no significant effect on COMMD1 mRNA levels, and that aspirin caused a decrease in COMMD1 mRNA, despite causing an increase in cellular levels of the protein (Fig. 2F). Hence, we concluded that p300 and aspirin influence COMMD1 stability.

In addition to regulation of NF-κB, COMMD1 has a number of other important functions. These include Cu^2+^ and Na^+^ transport, cell proliferation and adaptation to hypoxia (Bartuzi et al., 2013). In keeping with these roles, mice null for COMMD1 show embryonic lethality and dysregulation of COMMD1 is associated with diseases such as copper toxicosis and cancer (Li et al., 2014; Sarkar and Roberts, 2011; van de Sluis et al., 2010). Hence, pathways that control COMMD1 activity are extremely interesting. Identified mechanisms of regulation include cytoplasmic–nuclear shuttling, mRNA stability (Muller et al., 2007) and proteasomal degradation (Burstein et al., 2004). This is the first demonstration that COMMD1 is regulated by acetylation, and so warranted further investigation.

### Acetylation and deacetylation regulates XIAP-mediated degradation of COMMD1

**Fig. 3.**
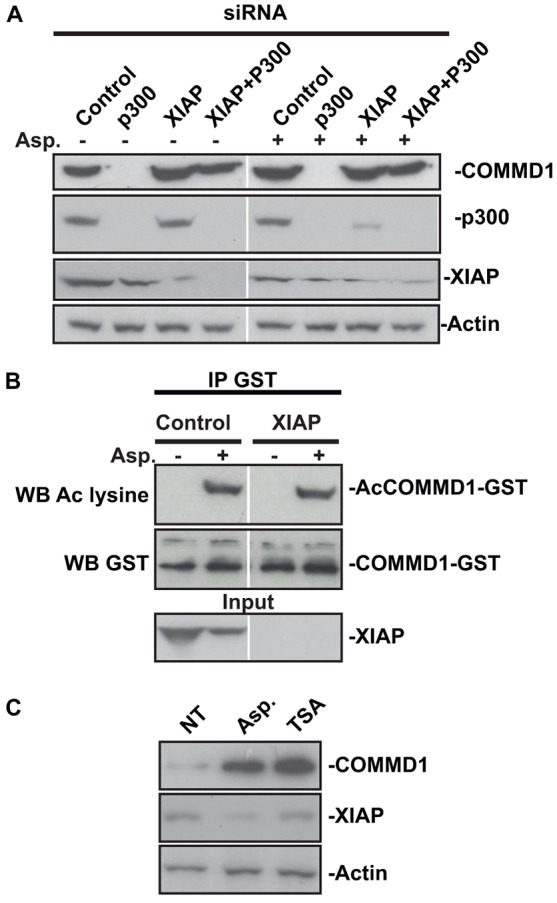
**COMMD1 acetylation and deacetylation protects against XIAP-mediated degradation.** (A) SW480 cells were transfected with control, p300 and XIAP siRNA, either individually or together. Western blotting (WB) was performed on whole cell lysates. Actin controlled for loading. COMMD1 levels are depleted by p300 knockdown but rescued by concomitant knockdown of XIAP. (B) Cells were transfected with GST–COMMD1 alongside control or XIAP siRNA. COMMD1 was isolated and the level of acetylated (Ac) COMMD1 was determined as in Fig. 2. Input levels of XIAP confirm knockdown. (C) Cells were not treated (NT) or treated with aspirin (5 mM) or trichostatin A (TSA, 400 nM) for 16 h. The western blot shows COMMD1 and XIAP levels.

Previous studies have indicated COMMD1 is ubiquitylated by the E3 ligase XIAP, then targeted for degradation (Mufti et al., 2007). Therefore, we next determined whether this pathway was involved in COMMD1 regulation by p300. Fig. 3A demonstrates that the reduction in COMMD1 observed upon loss of p300 is reversed by concomitant depletion of XIAP.

These exciting data would suggest that there is competition between COMMD1 acetylation and ubiquitylation. If so, we would expect acetylated COMMD1 levels to increase upon XIAP knockdown. However, XIAP depletion did not increase either basal or induced COMMD1 acetylation, despite increasing absolute COMMD1 levels (Fig. 3B). This finding suggests COMMD1 is both acetylated and deacetylated. Indeed, we found that the histone deacetylase inhibitor TSA, caused a dramatic increase in COMMD1 levels, which was independent of XIAP (Fig. 3C).

These data confirm that acetylation plays an important role in regulating COMMD1 stability and suggest that under basal conditions, COMMD1 is continuously acetylated and deactylated, which limits the rate of XIAP-mediated ubiquitylation and degradation.

### p300-mediated acetylation of COMMD1 is required for RelA binding in response to aspirin and is induced in a stimuli-specific manner

**Fig. 4.**
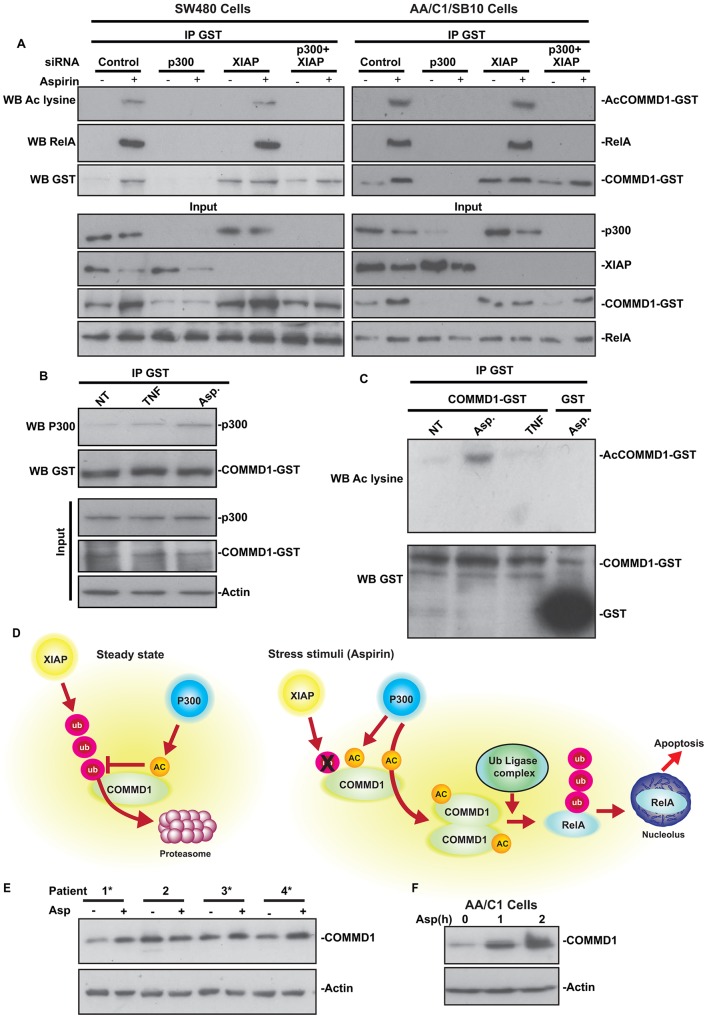
**p300-mediated acetylation of COMMD1 is stimuli specific and is required for RelA binding in response to stress.** (A–C) SW480 or AA/C1/SB10 cells were transfected with GST–COMMD1 alone (B,C) or with the siRNA species indicated (A). Cells were treated with aspirin (Asp) (10 mM) or TNF (10 ng/µl) for 2 h prior to harvest. GST–COMMD1 was immunoprecipitated (IP) as in Fig. 2, then isolated proteins were subjected to western blotting (WB) with the antibodies indicated. Input levels of the proteins of interest are shown. (D) Model for the role of acetylation in regulating the steady state levels of COMMD1 and the ubiquitylation and nucleolar translocation of RelA. See discussion for details. AC, acetylation; ub, ubiquitylation. (E) Tumour biopsies from four colorectal cancer patients were exposed to pharmacological doses of aspirin (100 µM, 1 h) *ex vivo* (see supplementary material Fig. S1). Western blot showing COMMD1 levels in whole-cell lysates. The asterisks indicate biopsies where COMMD1 increases in response to aspirin. (F) Western blot showing effects of aspirin (10 mM) on COMMD1 levels in the pre-tumorigenic AA/C1 cell line.

Throughout the course of these studies, we observed a reduction in XIAP in response to aspirin, which leads to increased amounts of COMMD1. Therefore, it could be argued that the effects of aspirin are dependent on XIAP and independent of p300. To further address the role of p300, we utilised the fact that knocking down p300 and XIAP together results in substantial levels of COMMD1 that, if p300 is the crucial acetyltransferase, should not be acetylated (Fig. 3A). We found that, as predicted, p300 loss abrogated aspirin-mediated COMMD1 acetylation (Fig. 4A). We also found that the interaction between RelA and COMMD1, detected in cells transfected with control and XIAP siRNA, was completely lost when p300 was depleted, despite substantial COMMD1 levels (see Fig. 4A, lane 8). These data, which were confirmed in an independent cell line (Fig. 4A), reveal that p300 is required for aspirin-mediated acetylation of COMMD1 and that this is essential for nucleolar translocation of RelA not only to stabilise COMMD1, but also to enable a RelA–COMMD1 interaction. In contrast to aspirin, we observed a minimal increase in COMMD1–p300 binding or in COMMD1 acetylation in response to TNF (Fig. 4B,C).

Taken together, our data suggest the model outlined in Fig. 4D. We suggest acetylation blocks XIAP-mediated ubiquitylation of COMMD1 and, consequently, the rate of acetylation and deacetylation which determines the steady-state levels of the protein. We propose that this dynamic is altered upon exposure to specific stress stimuli, resulting in increased COMMD1–p300 binding and increased levels of acetylated protein. We propose that the acetylated form of COMMD1 recruits a unique ubiquitin ligase complex that ubiquitylates RelA with a signature that drives it to the nucleolus to mediate apoptosis (Khandelwal et al., 2011), rather than to the proteasome.

In support of our suggestion that aspirin and TNF recruit different ubiquitin ligase complexes, it has been shown that phosphorylation at S468 (Geng et al., 2009; Mao et al., 2009) and methylation at K314 and K315 (Yang et al., 2009) are crucial for TNF-mediated ubiquitylation and degradation of RelA, whereas we have shown that deleting the C-terminus of RelA does not affect aspirin-mediated ubiquitylation or nucleolar translocation of the protein (our unpublished data; Stark and Dunlop, 2005). Furthermore, we found that calyculin A promotes S468 and/or S536 phosphorylation while blocking aspirin and MG132-mediated RelA ubiquitylation (Thoms et al., 2010). The ubiquitin ligase complex that ubiquitylates RelA in response to TNF, and the pattern of this ubiquitylation has been well defined (Li et al., 2012; Mao et al., 2009). Our data open up new avenues of research into the RelA ubiquitylation signature that targets the protein to the nucleolus, and the ubiquitin ligase complex associated with p300-acetylated COMMD1.

### Aspirin-mediated stabilisation of COMMD1 in biopsies of human tumours

Aspirin acts as a chemopreventative agent against colorectal cancer (Din et al., 2010). To determine the relevance of our findings to this activity, we treated biopsies of fresh colorectal tumours with pharmacological doses of the agent *ex vivo* (Fig. 4E; supplementary material Fig. S1). We found that 1 h aspirin (100 µM) treatment induced an increase in COMMD1 levels in three out of four tumours. We also found that aspirin caused an increase in COMMD1 levels in the pre-tumorigenic AA/C1 cell line (Fig. 4F) and its tumorigenic equivalent AA/C1/SB10 (Fig. 4A) (Williams et al., 1990). Based on these findings and our previous studies, we suggest that stabilisation of COMMD1, through p300-mediated acetylation, is important for the chemopreventative activity of the agent.

## MATERIALS AND METHODS

### Cell lines and reagents

Human SW480 colon cancer cells (American Type Culture Collection) and those expressing GFP–RelA (SW480-GFP-RelA) were grown as described previously (Thoms et al., 2010). AA/C1 and AAC1/SB10 cells were a gift from Ann C. Williams (School of Molecular and Cellular Medicine, University of Bristol, UK). Aspirin and trichostatin A (TSA) (Sigma) were prepared as described previously (Loveridge et al., 2008). TNF (R&D Systems) was used as specified in the text.

### Plasmids and transfections

The GFP–RelA expression construct was gifted by Eva Qwarnstrom (University of Sheffield, UK), the His_6_-Ubiquitin (Ub) plasmid by Ronald T. Hay (University of Dundee, UK) the GST–COMMD1 vector plus control by Erza Burstein (Ann Arbor Medical Centre, MI) (Maine et al., 2007) and the p300WT and ΔHAT plasmids by Kathryn Ball (University of Edinburgh, UK) (Dornan et al., 2004). siRNA species used were as follows: p300a (CAGAGCAGUCCUGGAUUAGUU), p300b (Santa Cruz Biotechnology) and XIAP (Santa Cruz Biotechnology). Plasmid and siRNA transfections were carried out using Lipofectamine 2000 (Gibco, BRL) according to the manufacturer's instructions.

### Immunoprecipitations

Immunoprecipitations for GST–COMMD1 and His_6_–Ub were performed on whole-cell lysates (Thoms et al., 2010).

### Western blot analysis

Whole-cell extracts were prepared and immunoblots performed on 30 µg of extract using standard procedures (Thoms et al., 2010). Primary antibodies were: rabbit anti-p65 (C-20) (Santa Cruz), mouse anti-p65 (Santa Cruz Biotechnology), rabbit anti-p65 (acetyl K310) (Abcam), mouse anti-His (generated in house), mouse anti-p300 (BD Pharmingen), rabbit anti-acetylated lysine (Cell Signaling), mouse anti-COMMD1 (Abnova), anti-XIAP (Santa Cruz) and anti-GST–HRP (Abcam) antibodies. Actin (Amersham) controlled for protein loading.

### Immunocytochemical staining

Cells were fixed in 1∶1 methanol∶acetone (−20°C), then immunocytochemistry was performed using standard procedures. Primary antibodies were against p65 (rabbit polyclonal, C20) and mouse monoclonal, C23 (both Santa Cruz Biotechnology). Fluorescent microscopy was performed with a Zeiss Axioplan microscope, a 63× Plan Neofluor objective and a Chroma 83,000 filter set. Images were captured using IPLab Spectrum 3.6. The percentage of cells showing nucleolar RelA was determined in >200 cells from more than five fields of view in three independent experiments or as specified.

### Quantitative PCR

Total RNA was isolated from cells using TRIzol (Invitrogen) then cDNA generated using a 1st Strand cDNA synthesis kit (Roche). The Roche TaqMan COMMD1 assay and the reference gene, GAPDH, were used with a LightCycler 480 system (Roche) to quantify cDNA. The Pfaffl method was used for relative quantitation of the target and reference gene transcripts.

### Site-directed mutagenesis and live-cell imaging

GFP–RelAWT was mutated at the specified lysine residues using the QuikChange site-directed mutagenesis kit (Stratagene) with specific primers. The sequence of all constructs was confirmed. An Axiovert 100 inverted microscope (Zeiss) determined the localisation of GFP-tagged proteins in live cells. Images were captured and processed using IPLab 3.6 with scripts written in-house.

### *Ex vivo* treatment of colorectal tumour biopsies

These studies were undertaken with patient consent and full ethical approval according to the Declaration of Helsinki (2000) (Scottish Colorectal Cancer Genetic Susceptibility Study 3; Reference, 11/SS/0109). Details in supplementary material Fig. S1.

## Supplementary Material

Supplementary Material

## References

[b1] Bartuzi P. Hofker M. H. van de Sluis B. (2013). Tuning NF-κB activity: a touch of COMMD proteins. Biochim. Biophys. Acta 1832, 2315–2321 10.1016/j.bbadis.2013.09.014 24080195

[b2] Burstein E. Ganesh L. Dick R. D. van De Sluis B. Wilkinson J. C. Klomp L. W. Wijmenga C. Brewer G. J. Nabel G. J. Duckett C. S. (2004). A novel role for XIAP in copper homeostasis through regulation of MURR1. EMBO J. 23, 244–254 10.1038/sj.emboj.7600031 14685266 PMC1271669

[b3] Chen L. F. Greene W. C. (2004). Shaping the nuclear action of NF-kappaB. Nat. Rev. Mol. Cell Biol. 5, 392–401 10.1038/nrm1368 15122352

[b4] Colleran A. Collins P. E. O'Carroll C. Ahmed A. Mao X. McManus B. Kiely P. A. Burstein E. Carmody R. J. (2013). Deubiquitination of NF-kappaB by ubiquitin-specific protease-7 promotes transcription. Proc. Natl. Acad. Sci. USA 110, 618–623 10.1073/pnas.1208446110 23267096 PMC3545798

[b5] Din F. V. Theodoratou E. Farrington S. M. Tenesa A. Barnetson R. A. Cetnarskyj R. Stark L. Porteous M. E. Campbell H. Dunlop M. G. (2010). Effect of aspirin and NSAIDs on risk and survival from colorectal cancer. Gut 59, 1670–1679 10.1136/gut.2009.203000 20844293

[b6] Dornan D. Eckert M. Wallace M. Shimizu H. Ramsay E. Hupp T. R. Ball K. L. (2004). Interferon regulatory factor 1 binding to p300 stimulates DNA-dependent acetylation of p53. Mol. Cell. Biol. 24, 10083–10098 10.1128/MCB.24.22.10083-10098.2004 15509808 PMC525491

[b7] Geng H. Wittwer T. Dittrich-Breiholz O. Kracht M. Schmitz M. L. (2009). Phosphorylation of NF-kappaB p65 at Ser468 controls its COMMD1-dependent ubiquitination and target gene-specific proteasomal elimination. EMBO Rep. 10, 381–386 10.1038/embor.2009.10 19270718 PMC2672889

[b8] Huang B. Yang X. D. Lamb A. Chen L. F. (2010). Posttranslational modifications of NF-kappaB: another layer of regulation for NF-kappaB signaling pathway. Cell. Signal. 22, 1282–1290 10.1016/j.cellsig.2010.03.017 20363318 PMC2893268

[b9] Karin M. (1999). The beginning of the end: IkappaB kinase (IKK) and NF-kappaB activation. J. Biol. Chem. 274, 27339–27342 10.1074/jbc.274.39.27339 10488062

[b10] Khandelwal N. Simpson J. Taylor G. Rafique S. Whitehouse A. Hiscox J. Stark L. A. (2011). Nucleolar NF-κB/RelA mediates apoptosis by causing cytoplasmic relocalization of nucleophosmin. Cell Death Differ. 18, 1889–1903 10.1038/cdd.2011.79 21660047 PMC3214916

[b11] Li H. Wittwer T. Weber A. Schneider H. Moreno R. Maine G. N. Kracht M. Schmitz M. L. Burstein E. (2012). Regulation of NF-kappaB activity by competition between RelA acetylation and ubiquitination. Oncogene 31, 611–623 21706061 10.1038/onc.2011.253 PMC3183278

[b12] Li H. Chan L. Bartuzi P. Melton S. D. Weber A. Ben-Shlomo S. Varol C. Raetz M. Mao X. Starokadomskyy P. (2014). Copper metabolism domain-containing 1 represses genes that promote inflammation and protects mice from colitis and colitis-associated cancer. Gastroenterology 147, 184–195.e3 24727021 10.1053/j.gastro.2014.04.007 PMC4086320

[b13] Loveridge C. J. MacDonald A. D. Thoms H. C. Dunlop M. G. Stark L. A. (2008). The proapoptotic effects of sulindac, sulindac sulfone and indomethacin are mediated by nucleolar translocation of the RelA(p65) subunit of NF-kappaB. Oncogene 27, 2648–2655 10.1038/sj.onc.1210891 18059344

[b100] Maine G. N. Mao X. Komarck C. M. Burstein E. (2007). COMMD1 promotes the ubiquitination of NF-kappaB subunits through a cullin-containing ubiquitin ligase. EMBO J. 26, 436–447 10.1038/sj.emboj.7601489 17183367 PMC1783443

[b14] Mao X. Gluck N. Li D. Maine G. N. Li H. Zaidi I. W. Repaka A. Mayo M. W. Burstein E. (2009). GCN5 is a required cofactor for a ubiquitin ligase that targets NF-kappaB/RelA. Genes Dev. 23, 849–861 10.1101/gad.1748409 19339690 PMC2666342

[b15] Mufti A. R. Burstein E. Duckett C. S. (2007). XIAP: cell death regulation meets copper homeostasis. Arch. Biochem. Biophys. 463, 168–174 10.1016/j.abb.2007.01.033 17382285 PMC1986780

[b16] Muller P. van Bakel H. van de Sluis B. Holstege F. Wijmenga C. Klomp L. W. (2007). Gene expression profiling of liver cells after copper overload in vivo and in vitro reveals new copper-regulated genes. J. Biol. Inorg. Chem. 12, 495–507 10.1007/s00775-006-0201-y 17211630

[b17] Perkins N. D. Gilmore T. D. (2006). Good cop, bad cop: the different faces of NF-kappaB. Cell Death Differ. 13, 759–772 10.1038/sj.cdd.4401838 16410803

[b18] Saccani S. Marazzi I. Beg A. A. Natoli G. (2004). Degradation of promoter-bound p65/RelA is essential for the prompt termination of the nuclear factor kappaB response. J. Exp. Med. 200, 107–113 10.1084/jem.20040196 15226358 PMC2213320

[b19] Sarkar B. Roberts E. A. (2011). The puzzle posed by COMMD1, a newly discovered protein binding Cu(II). Metallomics 3, 20–27 10.1039/c0mt00031k 21275100

[b20] Stark L. A. Dunlop M. G. (2005). Nucleolar sequestration of RelA (p65) regulates NF-kappaB-driven transcription and apoptosis. Mol. Cell. Biol. 25, 5985–6004 10.1128/MCB.25.14.5985-6004.2005 15988014 PMC1168799

[b21] Thoms H. C. Dunlop M. G. Stark L. A. (2007). p38-mediated inactivation of cyclin D1/cyclin-dependent kinase 4 stimulates nucleolar translocation of RelA and apoptosis in colorectal cancer cells. Cancer Res. 67, 1660–1669 10.1158/0008-5472.CAN-06-1038 17308107

[b22] Thoms H. C. Loveridge C. J. Simpson J. Clipson A. Reinhardt K. Dunlop M. G. Stark L. A. (2010). Nucleolar targeting of RelA(p65) is regulated by COMMD1-dependent ubiquitination. Cancer Res. 70, 139–149 10.1158/0008-5472.CAN-09-1397 20048074

[b23] van de Sluis B. Mao X. Zhai Y. Groot A. J. Vermeulen J. F. van der Wall E. van Diest P. J. Hofker M. H. Wijmenga C. Klomp L. W. (2010). COMMD1 disrupts HIF-1alpha/beta dimerization and inhibits human tumor cell invasion. J. Clin. Invest. 120, 2119–2130 10.1172/JCI40583 20458141 PMC2877941

[b24] Williams A. C. Harper S. J. Paraskeva C. (1990). Neoplastic transformation of a human colonic epithelial cell line: in vitro evidence for the adenoma to carcinoma sequence. Cancer Res. 50, 4724–4730 2369746

[b25] Yang X. D. Huang B. Li M. Lamb A. Kelleher N. L. Chen L. F. (2009). Negative regulation of NF-kappaB action by Set9-mediated lysine methylation of the RelA subunit. EMBO J. 28, 1055–1066 10.1038/emboj.2009.55 19262565 PMC2683704

[b26] Yang X. D. Tajkhorshid E. Chen L. F. (2010). Functional interplay between acetylation and methylation of the RelA subunit of NF-kappaB. Mol. Cell. Biol. 30, 2170–2180 10.1128/MCB.01343-09 20160011 PMC2863596

