# Supplementary material for: p300-mediated acetylation of COMMD1 regulates its stability, and the ubiquitylation and nucleolar translocation of the RelA NF-κB subunit
Source: J Cell Sci. 2014 Sep 1;127(17):3659–65. doi: 10.1242/jcs.149328 (PMC4150058; doi:10.1242/jcs.149328)
Supplement: Supplementary Material [file supp_127_17_3659__index.html]

p300-mediated acetylation of COMMD1 regulates its stability, and the ubiquitylation and nucleolar translocation of the RelA NF-κB subunit — Supplementary Material 

# p300-mediated acetylation of COMMD1 regulates its stability, and the ubiquitylation and nucleolar translocation of the RelA NF-κB subunit

## JCS149328 Supplementary Material

**Files in this Data Supplement:**

- **Supplementary Material**
