# Supplementary material for: p300-mediated acetylation of COMMD1 regulates its stability, and the ubiquitylation and nucleolar translocation of the RelA NF-κB subunit
Source: J Cell Sci. 2014 Sep 1;127(17):3659–65. doi: 10.1242/jcs.149328 (PMC4150058; doi:10.1242/jcs.149328)
Supplement: Supplementary Material [file supp_127.17.3659_JCS149328.pdf]

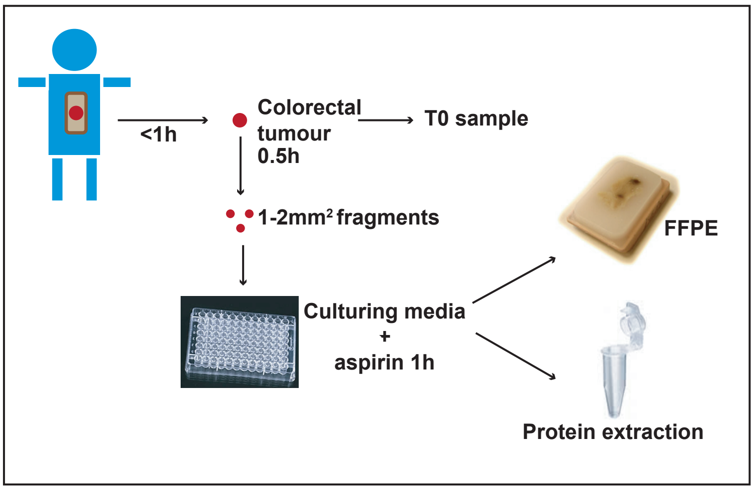

Supplemental figure 1. Aspiirin effects on COMMD1 levels in biopsies of human tumours. Biopsies of colorectal tumours were provided by a pathologist at the time of resection. All patients were consented and full ethical approval was in place (Scottish Colorectal Cancer Genetic Susceptibility Study 3; Reference: 11/SS/0109). Biopsies were immediately transferred to the lab immersed in culturing media (MEM supplemented with glutamine, penicillin/streptomycin and anti-mycotic/antibiotic mix (1:100, Sigma). Tumours were placed in Falcon tubes containing sterile PBS, washed twice by inverting the tubes, and then placed in Petri dishes containing sterile culturing media and dissected into 1-2mm2 fragments. They were then transferred to new Petri dishes and washed with fresh media before plating. Treatment (0-100μM aspirin, 1h, 37°C) of tumour explants was performed in 96-well plates and in the presence of 10% foetal calf serum. Whole cell extracts were prepared from tumour tissue using a TissueLyser (Qiagen) and standard whole cell lysis buffer.
